# Airway opening pressure maneuver to detect airway closure in mechanically ventilated pediatric patients

**DOI:** 10.3389/fped.2024.1310494

**Published:** 2024-02-06

**Authors:** Luciana Rodriguez Guerineau, Fernando Vieira, Antenor Rodrigues, Katherine Reise, Mark Todd, Anne-Marie Guerguerian, Laurent Brochard

**Affiliations:** ^1^ Department of Critical Care Medicine, Hospital for Sick Children, Interdepartmental Division of Critical Care Medicine, University of Toronto, Toronto, ON, Canada; ^2^ Keenan Centre for Biomedical Research, Li Ka Shing Knowledge Institute, St. Michael's Hospital, Interdepartmental Division of Critical Care Medicine, University of Toronto, Toronto, ON, Canada; ^3^ Department of Respiratory Therapy and Critical Care Medicine, Hospital for Sick Children, Toronto, ON, Canada

**Keywords:** airway closure, airway opening pressure maneuver, pediatrics, respiratory maneuvers, acute respiratory distress syndrome

## Abstract

**Background:**

Airway closure, which refers to the complete collapse of the airway, has been described under mechanical ventilation during anesthesia and more recently in adult patients with acute respiratory distress syndrome (ARDS). A ventilator maneuver can be used to identify airway closure and measure the pressure required for the airway to reopen, known as the airway opening pressure (AOP). Without that maneuver, AOP is unknown to clinicians.

**Objective:**

This study aims to demonstrate the technical adaptation of the adult maneuver for children and illustrate its application in two cases of pediatric ARDS (p-ARDS).

**Methods:**

A bench study was performed to adapt the maneuver for 3–50 kg patients. Four maneuvers were performed for each simulated patient, with 1, 2, 3, and 4 s of insufflation time to deliver a tidal volume (Vt) of 6 ml/kg by a continuous flow.

**Results:**

Airway closure was simulated, and AOP was visible at 15 cmH_2_O with a clear inflection point, except for the 3 kg simulated patient. Regarding insufflation time, a 4 s maneuver exhibited a better performance in 30 and 50 kg simulated patients since shorter insufflation times had excessive flowrates (>10 L/min). Below 20 kg, the difference in resistive pressure between a 3 s and a 4 sec maneuver was negligible; therefore, prolonging the maneuver beyond 3 s was not useful. Airway closure was identified in two p-ARDS patients, with the pediatric maneuver being employed in the 28 kg patient.

**Conclusions:**

We propose a pediatric AOP maneuver delivering 6 ml/kg of Vt at a continuous low-flow inflation for 3 s for patients weighing up to 20 kg and for 4 s for patients weighing beyond 20 kg.

## Introduction

The phenomenon of complete collapse of the airway, known as airway closure, was first described in 1967 using gas dilution techniques (1). It was mainly reported during anesthesia in adults (1, 2) and children (3, 4). The “classic” airway closure, as described by Hedenstierna (5), occurs in peripheral airways when the outside pressure (pleural pressure) is higher than the inside pressure. Due to the increase in pleural pressure caused by the weight of the lung, the dependent regions are surrounded by higher pressure and therefore prone to collapse during expiration; this is one of the major causes of atelectasis and hypoxemia during anesthesia (5). This mechanism of airway closure has been documented in rabbits with lung injuries using a synchrotron radiation technique in the distal airway (18th generation) (6). More recently, complete airway closure has been reported in up to 40% of adults with acute respiratory distress syndrome (ARDS) under mechanical ventilation, implying a more central location within the airway tree (7). The importance of this phenomenon lies in the fact that for gas to enter the lungs, the airway pressure needs to overcome the airway opening pressure (AOP). If airway pressure is below AOP, there is no communication with the distal alveoli, leading to denitrogenation atelectasis and making the measurement of driving pressure and calculations utilizing driving pressure inaccurate. A simple maneuver performed on the ventilator can identify the presence of AOP, without which it is unknown to clinicians (8). Designed for adults, the maneuver is a low-flow insufflation that, if airway closure is present, can differentiate between two different compliances: the ventilator's circuit (Ccircuit) and the patient's respiratory system (Crs) (8). The AOP will appear as an inflection point in the airway pressure curve (pressure–time curve) displayed on the ventilator, indicating the pressure level at which the airway reopens.

The presence of airway closure has not been described in children outside the context of anesthesia. We hypothesize that it occurs in children with severe pediatric ARDS (p-ARDS), given its prevalence in adults with ARDS. We report a bench study of the technical adaptation of the AOP maneuver to fit pediatric patients. We describe two cases of p-ARDS in which airway closure was present in the critical care setting. The first case prompted the adaptation of the AOP maneuver for pediatrics. In the second case, airway closure is documented using the adapted pediatric maneuver developed with the bench study.

## Methods

The bench study was designed to adapt the maneuver for patients with body weights ranging from 3 to 50 kg. A prototype was designed to simulate airway closure of 10 cmH_2_O above the set positive end-expiratory pressure (PEEP). We used a bidirectional PEEP valve (C-PEEP2210000, Intersurgical®, Workinham, UK) connected to a breathing simulator (ASL 5000TM IngMar^TM^ Medical, Pittsburgh, USA) (Supplementary Figure S1). The Servo-U (Getinge®, Goteborg, Sweden) mechanical ventilator was used during the simulated AOP maneuver. Breathing circuits and endotracheal tubes were selected according to size. The circuit was changed between the 30 kg and the 15 kg simulated patient from adult size (>20 kg) to neonatal size (<20 kg), following our institutional practice. The compliance of the circuit (Ccircuit) reported by the manufacturer is approximately 2.5 ml/cmH_2_O for the adult circuit and approximately 1.3 ml/cmH_2_O for the neonatal circuit. The circuit compliance compensation was set to off on the ventilator.

Four maneuvers were performed for each simulated body weight (3, 6, 15, 30, and 50 kg) with 1, 2, 3, or 4 s of insufflation time (Ti) to deliver a tidal volume (Vt) of 6 ml/kg by a continuous flow in SIMV volume control mode, with the respiratory rate set at 5 bpm and PEEP set at 5 cmH_2_O. We quantified the resistive pressure with an inspiratory hold of 0.3 s. The ServoTracker 4.1 recorded the ventilator data, while the ASL-5000 recorded the simulator data. We evaluated the technique to achieve a low-flow inflation maneuver able to identify airway closure, considering the following:

1) Insufflation time required to achieve a low-flow inflation curve with minimal resistive pressure
2) Visual identification of AOP in the pressure–time or pressure–volume (PV) curves
3) Ability to be replicated in all patients’ weights

The respiratory mechanics were set according to body weight (Supplementary Table S1). Two different sets of measurements were taken on a 3 kg patient using the Crs set in the simulator at 1 and 2 ml/cmH_2_O, which is similar to what is observed in neonates with lung problems. Since the circuit compliance (Ccircuit) is 1.3 ml/cmH_2_O, which is close to the compliance of a 3 kg patient, we simulated Crs for both scenarios below and above Ccircuit to assess the ability of the maneuver to identify AOP.

## Results

The description of results will adhere to the aforementioned criteria.

Resistive pressure at the tested insufflation times: The results are summarized in Table 1. In the 30 and 50 kg categories, the 4 s maneuver exhibited a better performance, similar to the adult maneuver with minimal resistive pressure. Shorter insufflation time maneuvers resulted in elevated flowrates (>10 L/min) and resistive pressure. Patients weighing less than 20 kg exhibited adequately low resistive pressure when the insufflation time was set to 3 s.

**Table 1:** Resistive pressure and flow with different insufflation times according to patient size.

Simulated patient				AOP maneuver							
				Ti = 4 s		Ti = 3 s		Ti = 2 s		Ti = 1 s	
Weight kg	Vt ml	Rrs cmH_2_O/L/sec	Crs ml/cmH_2_O	Rp cmH_2_O	Flow L/min	Rp cmH_2_O	Flow L/min	Rp cmH_2_O	Flow L/min	Rp cmH_2_O	Flow L/min
3	18	20	1 & 2	0.4	0.3	0.5	0.4	0.5	0.5	0.7	1.1
6	36	18	3	0.5	0.6	0.7	0.7	0.8	1.1	1.3	2.2
15	90	16	8	1.0	1.3	1.1	1.8	1.5	2.7	2.6	5.4
30	180	14	15	1.2	2.7	1.4	3.7	2.0	5.4	3.8	10.9
50	300	12	25	2.2	4.6	2.9	6.0	3.4	9.0	5.7	18.3

AOP, airway opening pressure; Ti, insufflation time; Vt, tidal volume; Rrs, respiratory system resistance of simulated patient; Crs, respiratory system compliance of simulated patient; Rp, resistive pressure.

**Figure 1:**
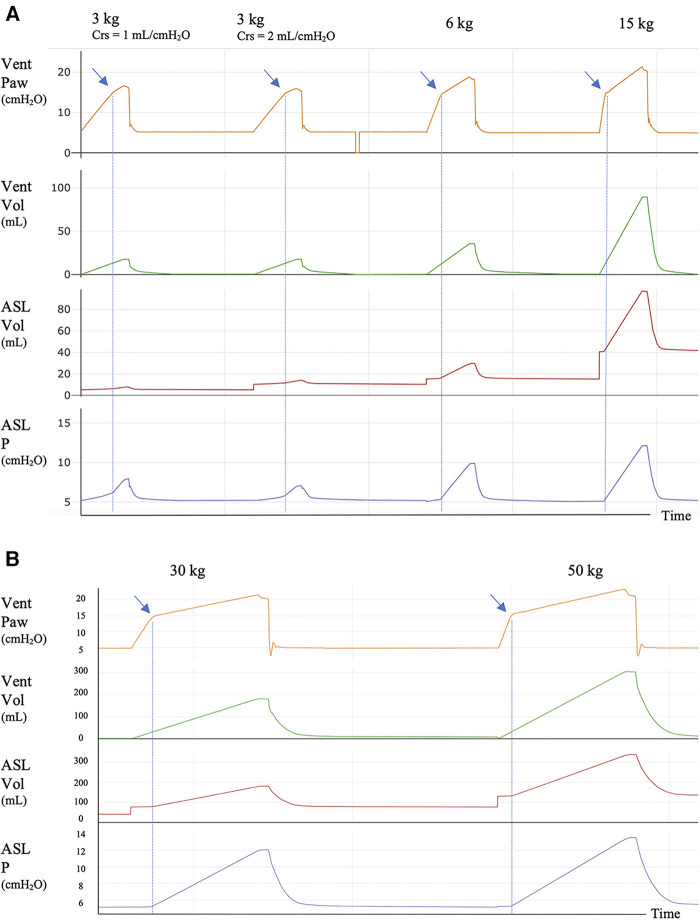
Ventilator and simulator tracings for 3, 6, and 15 kg simulated patients with 3 s of insufflation time and for 30 and 50 kg simulated patients with 4 s of insufflation time. (**A**) AOP maneuver for 3, 6, and 15 kg simulated patients with 3 s of insufflation time. (**B**) AOP maneuver for 30 and 50 kg simulated patients with 4 s of insufflation time. Crs: compliance of the respiratory system of the simulated patient. In the Y-axis: Vent Paw: airway pressure recorded in the ventilator; Vent Vol: volume recorded in the ventilator; ASL Vol: volume in the ASL simulator; ASL P: pressure in the ASL simulator. In the Vent Paw tracing, the AOP is seen as an inflection point (blue arrows) between two systems with different compliance, the circuit and the simulator. AOP is clearly identified in all patient sizes except for 3 kg simulated patients. In this category, two Crs were set, 1 and 2 ml/cmH_2_O, to reflect the range of compliance of the respiratory system that can be found in a 3 kg patient with lung injury. In both cases, the inflection point is difficult to be visualized [(**A**) blue arrows]. The circuit compliance of the 3 kg patient was 1.3 ml/cmH_2_O. The blue dotted lines mark the AOP; after this point, there is gas entry into the simulator with the consequent increase in volume and pressure. At the end of the maneuver, a short pause of 0.3 s was added to quantify the resistive pressure. There is no need to add this pause when the maneuver is performed in the clinical setting.

Visual identification of AOP: As depicted in Figure 1 (Supplementary Figures S2–S7 in all tested conditions), the AOP was approximately 15 cmH_2_O, which aligns with the bidirectional PEEP valve set at 10 cmH_2_O above the designated PEEP of 5 cmH_2_O, showing a clear inflection point, except for the 3 kg simulated patients.

**Figure 2:**
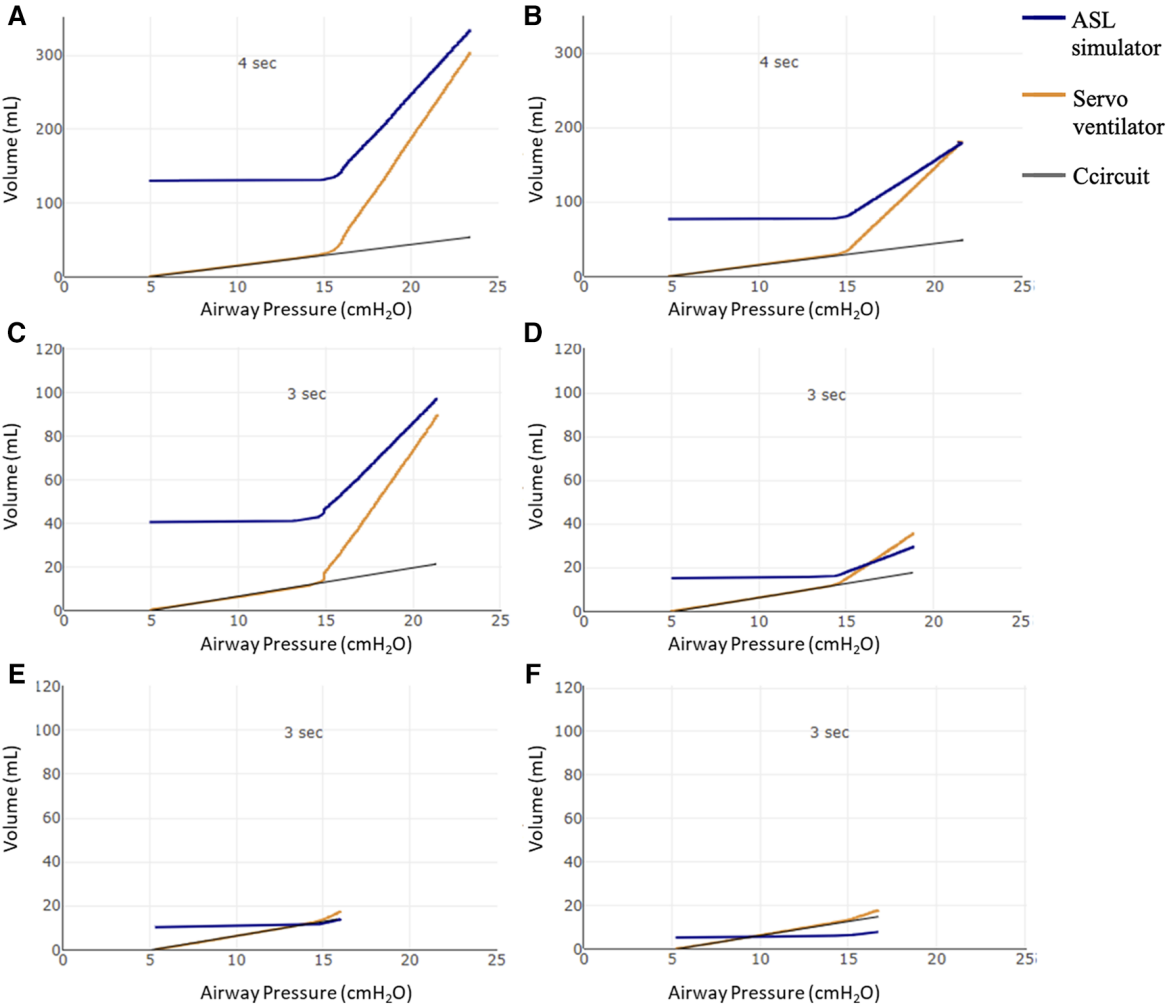
Pressure–volume curves using the proposed AOP maneuver. Volume (ml) in the Y-axis: starting from the PEEP volume (above functional residual capacity—FRC), the volume variation in the ASL simulator (blue line); the volume delivered by the Servo ventilator (orange line), Ccircuit: compliance of circuit (gray line). X axis: airway pressure in cmH_2_O. Pressure–volume curves using the proposed AOP maneuver with 4 s of insufflation time (Ti) in (**A**) 50 kg and (**B**) 30 kg patients. (**C–F**) 3 s maneuvers for 15, 6, and 3 kg—Crs = 2 ml/cmH_2_O and 3 kg—Crs = 1 ml/cmH_2_O patients, respectively. As expected, the pressure and volume on the ventilator (orange line) follows the circuit pressure (black line) until AOP is overcome. At that moment, when airway pressure is >15 cmH_2_O, air begins to enter the ASL simulator lung (blue line volume increases from PEEP volume) with a clear inflection point in the airway pressure seen in the ventilator (orange). In the 3 kg simulated patients (**E**,**F**), the inflection point is not as clear as it is seen in the other patient sizes.

AOP was also visualized in the pressure–volume curves as displayed in Figure 2.

Finally, for the reproducibility across all patient weights, two Ti settings are proposed to simplify the maneuver adequately for the pediatric critical care environment (3–50 kg). Simplifying the maneuver to two insufflation times is a pragmatic approach for clinicians to implement the technique at the patient’s bedside. While the adult maneuver (8) utilizes a fixed flowrate to achieve the desired flow and uses a variable Ti, the pediatric adaption involves using different flowrates based on the individual’s weight, determined by a fixed Ti and set Vt.

More details of the maneuvers and results per patient's size are shown in the Supplementary Figures S2–S7.

As a second step and proof of concept, we used the maneuver in the clinical setting. The standard operating procedure is detailed in the Supplementary Table S2. In the following section, we describe two pediatric cases in which airway closure was documented.

### Case report #1

**Figure 3:**
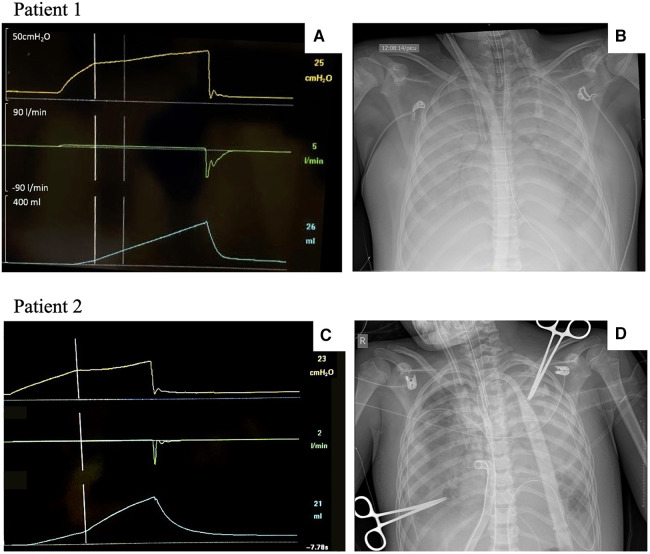
AOP maneuvers and chest x-rays in patients with p-ARDS. AOP, airway opening pressure; p-ARDS, pediatric acute respiratory distress syndrome. Patient 1: Adult AOP maneuver and chest x-ray in a 12-year-old patient with severe p-ARDS (**A**,**B**). (**A**) AOP maneuver with a clear identification of complete airway closure and an AOP at 25 cmH_2_O on Day 1 after ECMO cannulation. (**B**) Chest x-ray following cannulation. Patient 2: Adapted AOP maneuver and chest x-ray in a 9-year-old patient with severe p-ARDS (**C**,**D**). (**C**) Adapted AOP maneuver showing airway closure and an AOP at 23 cmH_2_O on Day 3 after ECMO cannulation. (**D**) Chest x-ray on the same day of the maneuver.

A previously healthy 12-year-old adolescent, weighing 65 kg (height 173 cm, predicted body weight 69 kg), was admitted to the PICU with severe p-ARDS. Due to refractory respiratory failure with conventional therapies, the patient was cannulated with veno-venous ECMO (VV ECMO) on Day 1. An AOP maneuver was performed following the adult guidelines (9) using a tidal volume of 6 ml/kg delivered in a low-flow (5 L/min) inflation after a prolonged expiration (respiratory rate of 5 bpm), which identified an AOP of 25 cmH_2_O (Figure 3A). The compliance of the respiratory system (Crs) was 14 ml/cmH_2_O, and the circuit compliance was 1.3 ml/cmH_2_O, similar to what was described by the manufacturer (Neonatal circuit due to extreme low Vt on ECLS). On Day 2 of ECMO, the AOP was 27 cmH_2_O with a Crs of 3.4 ml/cmH_2_O. Ventilation settings were pressure control (PC) 10–12 cmH_2_O above PEEP, with PEEP titrated from 10 to 15 cmH_2_O. From Day 3 onwards, ventilating pressures were below AOP (both peak inspiratory pressure and PEEP). The chest x-ray evolved with complete whiteout of both lungs (Figure 3B), and subsequent AOP maneuvers were unable to identify an inflection point, while the Crs were consistently below 5 ml/cmH_2_O. Progressive improvement of the Crs began during Week 5. The patient was decannulated successfully on Day 53 of VV ECMO support. The underlying etiology of p-ARDS was hemophagocytic lymphohistiocytosis without a precipitating factor.

### Case report #2

A previously healthy 9-year-old child, weighing 28 kg (height 130 cm, predicted body weight 25 kg), presented to an outside hospital with a 1-week history of feeling unwell. The child initially complained of pharyngitis, otalgia, and fever, which were followed by abdominal pain and vomiting. The patient was admitted to the pediatric ward for treatment of asthma and pneumonia. The clinical condition worsened with a left lung whiteout, pleural effusion on chest x-ray, increased work of breathing, and concerns for sepsis, for which the patient received fluid resuscitation, inotropic and vasoactive support, and finally required intubation. After intubation and on conventional mechanical ventilation, the patient continued to be hypoxemic despite recruitment maneuvers, the addition of inhaled nitric oxide, and a brief trial of high-frequency oscillatory ventilation. The patient evolved with features of refractory septic shock and severe p-ARDS, for which they were centrally cannulated onto a veno-arterial ECMO (VA ECMO) for cardiopulmonary support. The skin was closed over the sternotomy. The microbiology analysis of the pleural fluid and bronchoalveolar lavage revealed the presence of group A *Streptococcus*. The ventilation settings over the first 72 h involved using PC with a range of 10–14 cmH_2_O above a PEEP of 10–12 cmH_2_O with a progressive deterioration in Crs during this period. The adapted AOP 4 s maneuver was performed on ECMO Day 3 (Crs 12 ml/cmH_2_O) and showed airway closure with an AOP of 23 cmH_2_O (Figure 3C). The clinical status worsened despite a multidisciplinary approach to treat the infection and modulate the immune response, and the patient died on Day 6 of ECMO after withdrawal of life-sustaining therapies.

## Discussion

The aim of this report was to describe the technical adaptation of the AOP maneuver to accommodate pediatric-sized patients. The main findings indicate that (1) the pediatric AOP maneuver technique was successfully demonstrated in a bench simulation setting when 6 ml/kg of Vt was delivered by a continuous low-flow insufflation for 3 s (patients weighing up to 20 kg) and for 4 s (>20 kg); (2) the low-flow insufflation is accomplished by using a long Ti; and (3) the interpretation of the results may be limited when the patient's Crs is close to the circuit's compliance, such as in infants weighing approximately 3 kg with lung disease. In these circumstances, there is a possibility that it may not be reliable, and the subtle alteration in the pressure slope after the AOP occurrence may not be easily detected.

Airway closure was first described during anesthesia in adults (1, 2) and children (3, 4) using gas dilution techniques. More recently, Chen et al. (8) were able to demonstrate airway closure and quantify AOP in adult patients with ARDS by the use of a low-flow insufflation bedside maneuver. The pediatric adaptation of the maneuver adhered to the same principles. Similarly, the slope preceding the inflection point represents the compliance of the ventilator's circuit (see Supplementary Figures S2–S7). The inflection point corresponds to the pressure at which the airway opens (AOP), and after AOP, the slope follows the Crs of the patient. Decreasing the flow during the maneuver is essential to identify the inflection point, which indicates the opening of closed airways, while minimizing resistive pressure. It is important to note that resistive pressure can lead to an overestimation of the true AOP, as resistive pressure refers to the additional pressure displayed by the ventilator. Therefore, using a low-flow inflation method ensures minimal resistive pressure, facilitating the precise identification of the inflection point and preventing an overestimation of the true AOP, even in the setting of increased airway resistance. While the adult maneuver fixes inspiratory flow and has variable Ti (see video on https://respiratorycalc.com/ri-ratio), this pediatric adaption will have different flowrates based on the weight determined by the fixed Ti and set Vt in ml/kg, to facilitate reproducibility across the pediatric population. For example, following the standard operating procedure described in Supplementary Table S2, a 10 kg patient will get 60 ml of Vt over 3 s, which gives a flowrate of 1.2 L/min.

Airway closure is an increasingly recognized phenomenon in patients with obesity (10), ARDS (up to 40% of adult patients and 65% in those with body mass index of ≥40 kg/m^2^) (7), and more recently in patients with hydrostatic pulmonary edema caused by cardiogenic shock (11). The prevalence in pediatrics is unknown; however, with the use of this adapted maneuver, it is now possible to study its prevalence in pediatric patients with ARDS or other lung diseases. As demonstrated, we identified complete airway closure in two pediatric cases with severe ARDS on ECMO among a wider range of children with lung disease in whom the adapted maneuver was used with no inflection point visualized.

Previous studies have suggested a U-shape relationship of closing volume (the lung volume at which the airways start closing) and age, and therefore implying an increased risk of airway closure in younger children and in older adults: “A 7-year-old child may be at similar risk to a 45-year-old adult” according to Mansell et al. (3) with the lowest risk in the adolescent period. The authors postulated that this relationship follows the changes in lung elastic recoil with lung maturation. An increase in the amount and distribution of elastic tissue is seen during childhood, reaching their peak during the teenage years (12) followed by a gradual loss with age (13). The closing capacity (closing volume + residual volume) increases with age (14). In addition, functional residual capacity (FRC) is known to be lower in obese patients compared with non-obese patients (14). As a result, airway closure is expected to occur more often at a very young age, in patients with obesity and patients with ARDS (15). Therefore, its routine assessment has been recommended in patients with obesity and ARDS (10). Several causal factors have been postulated to contribute to airway closure in ARDS, including surfactant impairment, intraluminal fluid accumulation, bronchoconstriction, and the loss of radial traction secondary to elastic fiber destruction, with consequently decreased lung elastic recoil (15). In this clinical setting, if airway closure is present, the repeated opening and closing of the airway would further increase inflammation and contribute to ventilator-induced lung injury (16). Additionally, airway closure will promote atelectasis and ventilation perfusion mismatch and setting PEEP at or above AOP makes physiological sense and has been recommended (11, 17). For all these reasons, assessing airway closure and estimating AOP will help understand the pathophysiology of p-ARDS and other pediatric conditions, such as small airway disease, where airway closure may exist but has not been investigated. Future studies will be needed to further elucidate the prevalence and implications of airway closure in children with lung disease.

The main limitation of this maneuver is its interpretation when the compliance of the circuit and the patient's respiratory system are similar, and this situation can be common in small pediatric patients with lung disease.

## Conclusion

The presence of airway closure may occur in p-ARDS, and an adaptation of the maneuver was needed for pediatric patients. We propose a pediatric AOP maneuver developed in a bench simulation setting when 6 ml/kg of Vt was delivered using a continuous and low-flow insufflation for a duration of 3 s for patients weighing up to 20 kg and 4 s for patients weighing >20 kg. Caution should be taken when interpreting the results of the maneuver when the patient's Crs is close to the circuit’s compliance, such as in infants weighing approximately 3 kg with lung disease. The study demonstrated airway closure in two cases of severe p-ARDS requiring ECMO; the pediatric adaptation of the maneuver detected the airway closure and measured the AOP in a 28 kg patient.

## Data Availability

The original contributions presented in the study are included in the article/Supplementary Material; further inquiries can be directed to the corresponding author.
