# Supplementary material for: Airway opening pressure maneuver to detect airway closure in mechanically ventilated pediatric patients
Source: Front Pediatr. 2024 Feb 6;12:1310494. doi: 10.3389/fped.2024.1310494 (PMC10877025; doi:10.3389/fped.2024.1310494)
Supplement: Supplementary file 1 [file Datasheet1.pdf]

Supplementary Material

Airway opening pressure maneuver to detect airway closure in mechanically ventilated pediatric patients

Luciana Rodriguez Guerineau*, Fernando Vieira, Antenor Rodrigues, Katherine Reise, Mark Todd, Anne-Marie Guerguerian, Laurent Brochard

*** Correspondence:** Corresponding Author: luciana.rodriguezguerineau@sickkids.ca

Figure 1: Simulator set up.

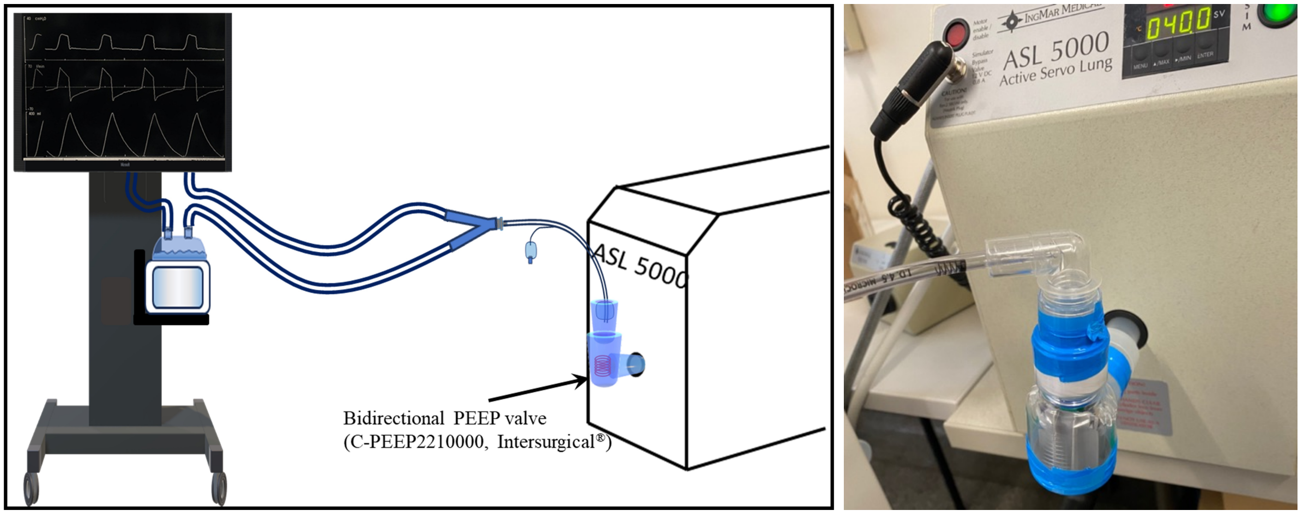

Figure 1: ASL 5000 simulator with a bidirectional PEEP valve before the connection of a size appropriate endotracheal tube (in the picture 4.5) to simulate airway closure of 10 cmH_2O above the set PEEP.

Table 1: Respiratory mechanics set in the ASL simulator and ETT size.

Size - Weight	FRC mL	Vt 6mL/kg mL	Rrs $cmH_2O/L/sec$	Crs mL/cmH_2O	ETT size
**3 kg**	100*	18	20*	1 & 2	3.0
**6 kg**	250	36	18	3	3.5
**15 kg**	500	90	16	8	4.5
**30 kg**	1000	180	14	15	5.5
**50 kg**	1500	300	12	25	7

*(14) Airway resistance and lung volume in the newborn infant. Pediatr Res. 1969;3(2):128-34. FRC: Functional residual capacity. Vt: Tidal volume. Rrs: Resistance of the respiratory system. Crs: Compliance of the respiratory system. ETT: Endotracheal tube.

Results per simulated patient weight

Figure 2: Ventilator and simulator tracings for 50 kg simulated patient

Figure 3: Ventilator and simulator tracings for 30 kg simulated patient

Figure 4: Ventilator and simulator tracings for 15 kg simulated patient

Figure 5: Ventilator and simulator tracings for 6 kg simulated patient

Figure 6: Ventilator and simulator tracings for 3 kg simulated patient / Crs=2 mL/cmH_2O

Figure 7: Ventilator and simulator tracings for 3 kg simulated patient / Crs=1 mL/cmH_2O

Figure 2: Ventilator and simulator tracings for 50 kg simulated patient with different insufflation times: from 4 sec to 1 sec. $C_{circuit}$ ~ 2.5 mL/cmH_2O

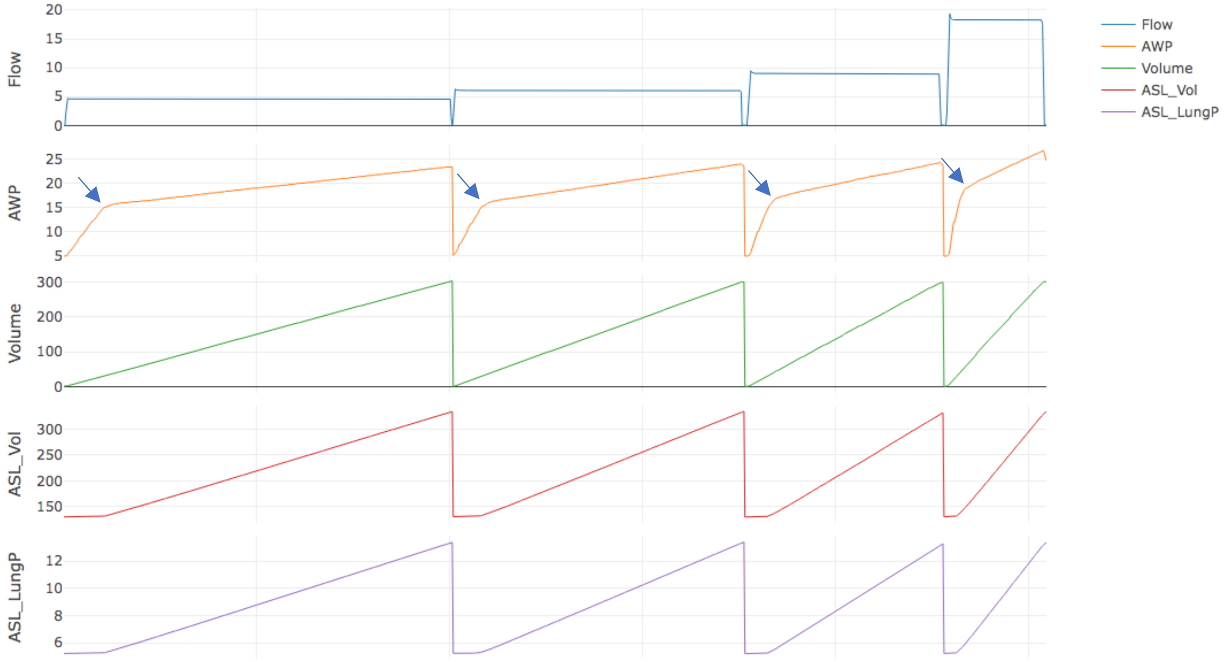

	4 sec maneuver	3 sec maneuver	2 sec maneuver	1 sec maneuver
Flow (L/min)	4.56	6.03	9	18.26
Delta-P_AOP (cmH_2O)	10	10.1	11.3	9.4
Delta-V_AOP (mL)	30.2	31.2	38.2	54.6
Ccircuit (mL/cmH_2O)	3.02	3.09	3.38	5.81
Delta-P_Patient (cmH_2O)	7.7	8	7.4	7.6
Delta-V_Patient (mL)	262.6	262.4	252.8	237.2
Crs (mL/cmH_2O)	34.10	32.80	34.16	31.21

In the Y axis: Flow, AWP: airway pressure and Volume recorded in the ventilator, ASL_Vol: Volume in the ASL simulator, ASL_LungP: Pressure in the simulator lung. In the AWP tracing the AOP is seen as an inflection point (blue arrows) at 15 cmH_2O.
Flow in L/min. Delta-P_AOP: Airway pressure from PEEP until AOP. Delta-V_AOP: Volume inside the circuit before AOP. Ccircuit: circuit compliance. Delta-P_Patient: Airway pressure from AOP to the end of the maneuver. Delta-V_Patient: Volume delivered from AOP until the end of the maneuver. Crs: Compliance of the simulated respiratory system.

Figure 3: Ventilator and simulator tracings for 30 kg simulated patient with different insufflation times: from 4 sec to 1 sec. $C_{circuit}$ ~ 2.5 mL/cmH_2O

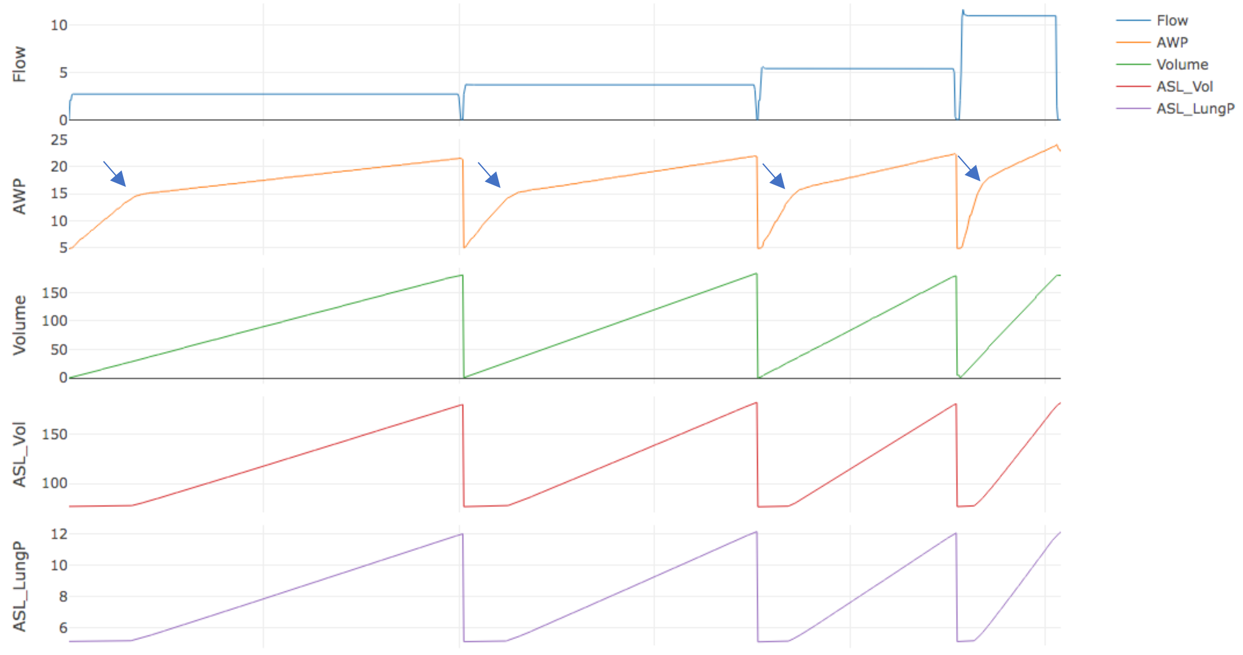

	4 sec maneuver	3 sec maneuver	2 sec maneuver	1 sec maneuver
Flow (L/min)	2.72	3.69	5.39	10.96
Delta-P_AOP (cmH_2O)	9.4	9.2	10.1	9.6
Delta-V_AOP (mL)	29	27.8	31.4	29.4
Ccircuit (mL/cmH_2O)	3.09	3.02	3.11	3.06
Delta-P_Patient (cmH_2O)	6.8	6.8	6.7	6.7
Delta-V_Patient (mL)	148	148	141.6	133
Crs (mL/cmH_2O)	21.76	21.76	21.13	19.85

In the Y axis: Flow, AWP: airway pressure and Volume recorded in the ventilator, ASL_Vol: Volume in the ASL simulator, ASL_LungP: Pressure in the simulator lung. In the AWP tracing the AOP is seen as an inflection point (blue arrows) at 15 cmH_2O.
Flow in L/min. Delta-P_AOP: Airway pressure from PEEP until AOP. Delta-V_AOP: Volume inside the circuit before AOP. Ccircuit: circuit compliance. Delta-P_Patient: Airway pressure from AOP to the end of the maneuver. Delta-V_Patient: Volume delivered from AOP until the end of the maneuver. Crs: Compliance of the simulated respiratory system.

Figure 4: Ventilator and simulator tracings for 15 kg simulated patient with different insufflation times: from 4 sec to 1 sec. $C_{circuit}$ ~ 1.3 mL/cmH_2O

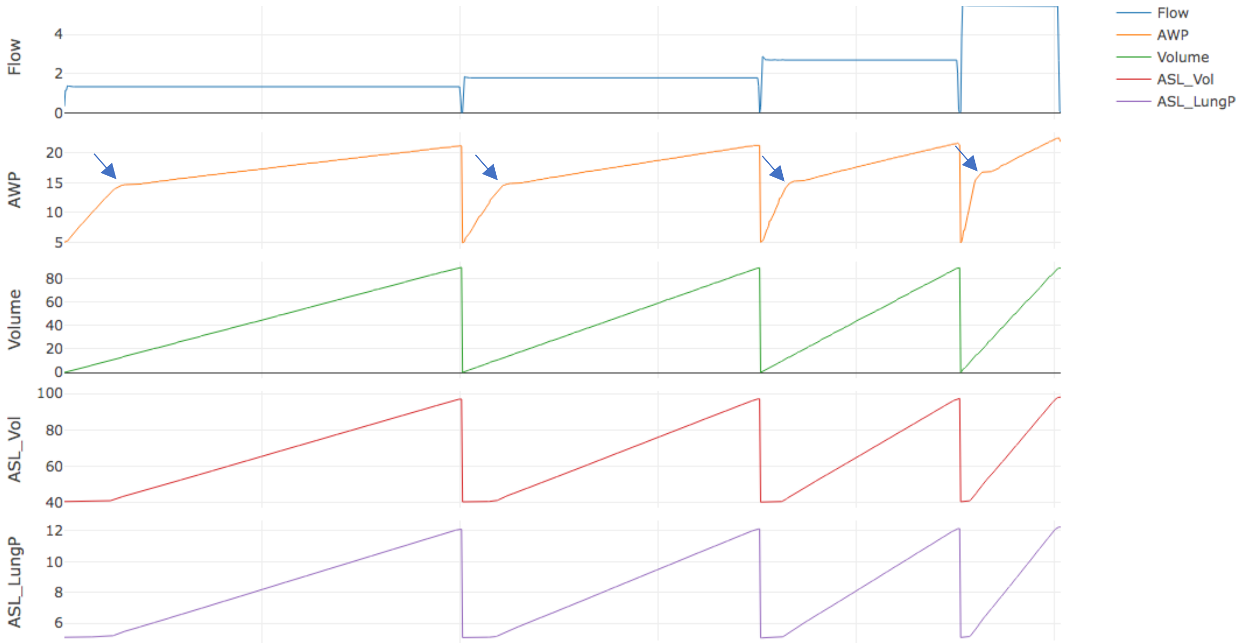

	4 sec maneuver	3 sec maneuver	2 sec maneuver	1 sec maneuver
Flow (L/min)	1.35	1.8	2.7	5.43
Delta-P_AOP (cmH_2O)	8.7	9.6	9.7	10.6
Delta-V_AOP (mL)	10.6	12.4	12.2	14.4
Ccircuit (mL/cmH_2O)	1.22	1.29	1.26	1.36
Delta-P_Patient (cmH_2O)	6.5	6.4	6.4	5.7
Delta-V_Patient (mL)	75.8	72.2	70	69
Crs (mL/cmH_2O)	11.66	11.28	10.94	12.11

In the Y axis: Flow, AWP: airway pressure and Volume recorded in the ventilator, ASL_Vol: Volume in the ASL simulator, ASL_LungP: Pressure in the simulator lung. In the AWP tracing the AOP is seen as an inflection point (blue arrows) at 15 cmH_2O.
Flow in L/min. Delta-P_AOP: Airway pressure from PEEP until AOP. Delta-V_AOP: Volume inside the circuit before AOP. Ccircuit: circuit compliance. Delta-P_Patient: Airway pressure from AOP to the end of the maneuver. Delta-V_Patient: Volume delivered from AOP until the end of the maneuver. Crs: Compliance of the simulated respiratory system.

Figure 5: Ventilator and simulator tracings for 6 kg simulated patient with different insufflation times: from 4 sec to 1 sec. $C_{circuit}$ ~ 1.3 mL/cmH_2O

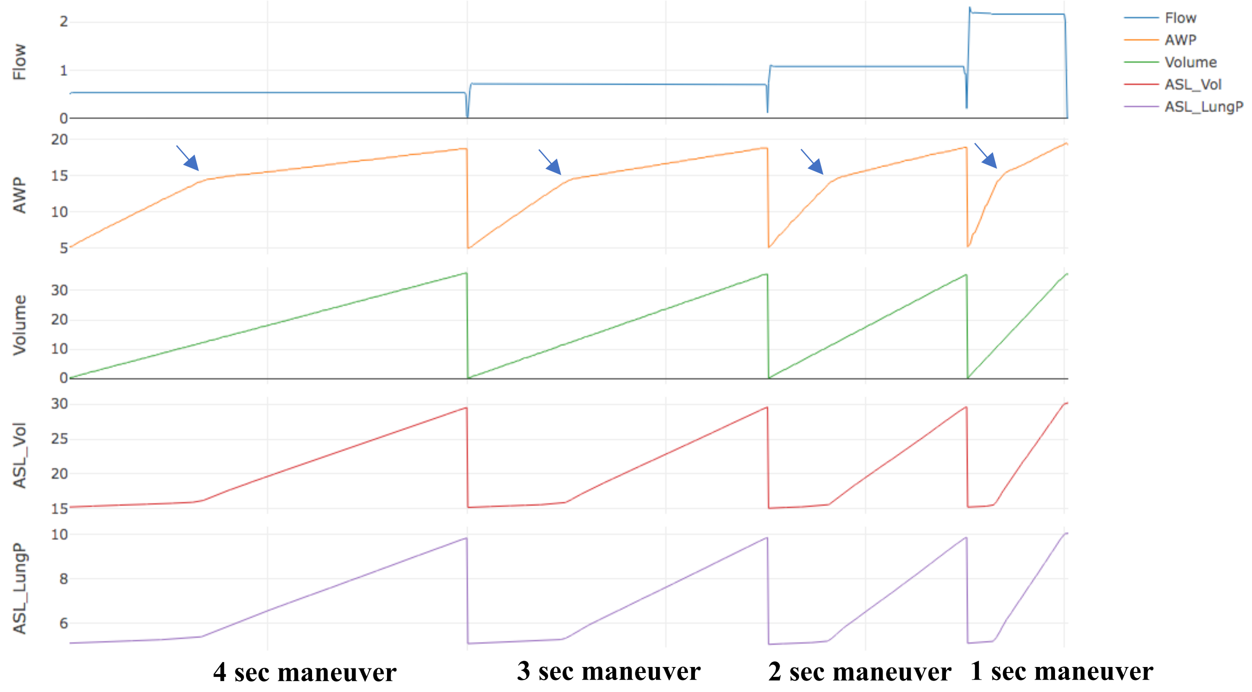

Flow (L/min)	0.6	0.72	1.08	2.16
Delta-P_AOP (cmH_2O)	9.3	9.1	8.9	9.7
Delta-V_AOP (mL)	12.2	11.4	11	12.4
Ccircuit (mL/cmH_2O)	1.31	1.25	1.24	1.28
Delta-P_Patient (cmH_2O)	4.1	4.2	4.2	3.7
Delta-V_Patient (mL)	23	22.8	22.6	20.2
Crs (mL/cmH_2O)	5.61	5.43	5.38	5.46

In the Y axis: Flow, AWP: airway pressure and Volume recorded in the ventilator, ASL_Vol: Volume in the ASL simulator, ASL_LungP: Pressure in the simulator lung. In the AWP tracing the AOP is seen as an inflection point (blue arrows) at 15 cmH_2O.
Flow in L/min. Delta-P_AOP: Airway pressure from PEEP until AOP. Delta-V_AOP: Volume inside the circuit before AOP. Ccircuit: circuit compliance. Delta-P_Patient: Airway pressure from AOP to the end of the maneuver. Delta-V_Patient: Volume delivered from AOP until the end of the maneuver. Crs: Compliance of the simulated respiratory system.

Figure 6: Ventilator and simulator tracings for 3 kg simulated patient / Crs=2 mL/cmH$_2$O with different insufflation times: from 4 sec to 1 sec. $C_{circuit}$ ~ 1.3 mL/cmH$_2$O

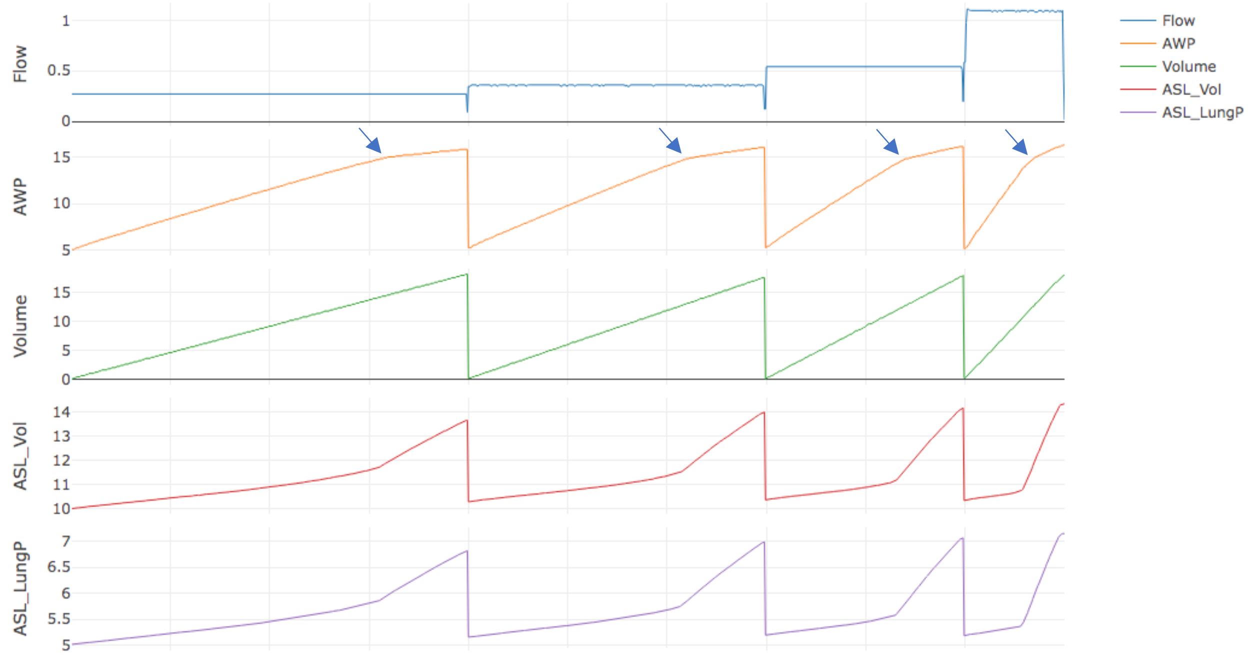

	4 sec maneuver	3 sec maneuver	2 sec maneuver	1 sec maneuver
Flow (L/min)	0.27	0.36	0.54	1.1
Delta-P_AOP (cmH$_2$O)	9.8	9.6	9.5	9.9
Delta-V_AOP (mL)	14.4	13.6	12.8	12.6
Ccircuit (mL/cmH$_2$O)	1.47	1.42	1.35	1.27
Delta-P_Patient (cmH$_2$O)	0.8	1.2	1.3	1.2
Delta-V_Patient (mL)	3.6	4.2	5	5
Crs (mL/cmH$_2$O)	4.50	3.50	3.85	4.17

In the Y axis: Flow, AWP: airway pressure and Volume recorded in the ventilator, ASL_Vol: Volume in the ASL simulator, ASL_LungP: Pressure in the simulator lung. In the AWP tracing the AOP can be identified at 15 cmH$_2$O (blue arrows) but it is not as clear as the previous examples. Flow in L/min. Delta-P_AOP: Airway pressure from PEEP until AOP. Delta-V_AOP: Volume inside the circuit before AOP. Ccircuit: circuit compliance. Delta-P_Patient: Airway pressure from AOP to the end of the maneuver. Delta-V_Patient: Volume delivered from AOP until the end of the maneuver. Crs: Compliance of the simulated respiratory system.

Figure 7: Ventilator and simulator tracings for 3 kg simulated patient / Crs=1 mL/cmH_2O with different insufflation times: from 4 sec to 1 sec. $C_{circuit}$ ~ 1.3 mL/cmH_2O

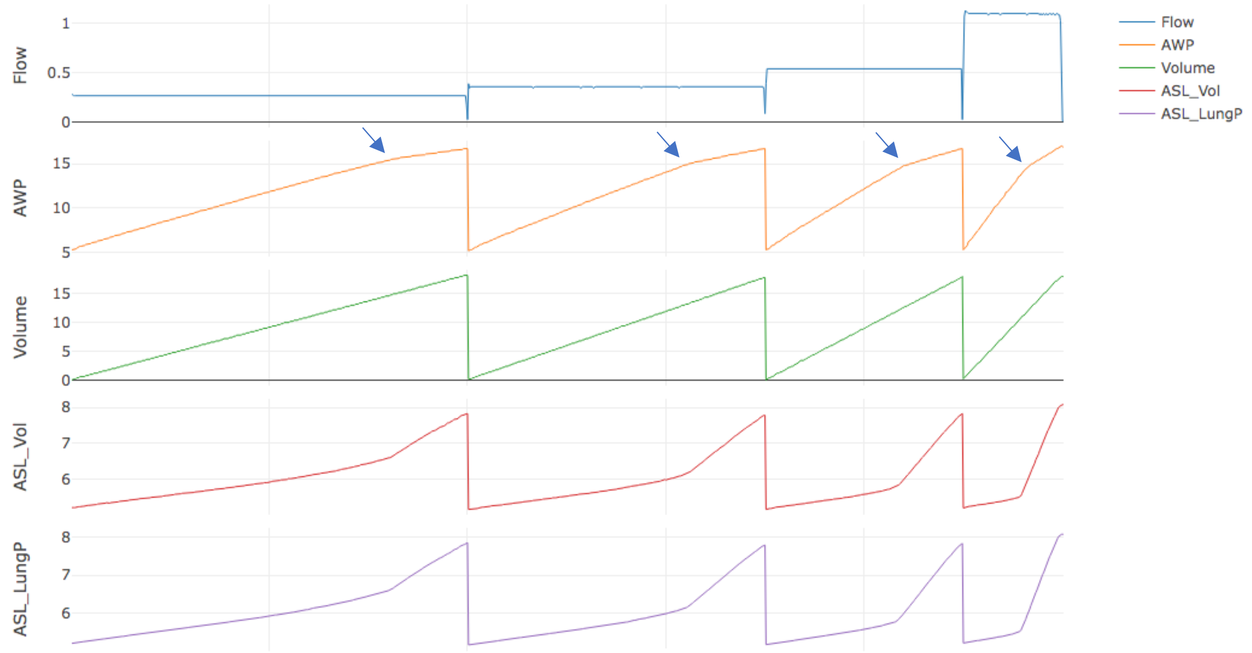

	4 sec maneuver	3 sec maneuver	2 sec maneuver	1 sec maneuver
Flow (L/min)	0.27	0.36	0.54	1.1
Delta-P_AOP (cmH_2O)	10.2	9.8	9.5	9.5
Delta-V_AOP (mL)	14.8	13.4	12.6	12.2
Ccircuit (mL/cmH_2O)	1.45	1.37	1.33	1.28
Delta-P_Patient (cmH_2O)	1.1	1.6	1.6	2
Delta-V_Patient (mL)	3.2	4.2	4.4	5.4
Crs (mL/cmH_2O)	2.91	2.63	2.75	2.70

In the Y axis: Flow, AWP: airway pressure and Volume recorded in the ventilator, ASL_Vol: Volume in the ASL simulator, ASL_LungP: Pressure in the simulator lung. In the AWP tracing the AOP can be identified at 15 cmH_2O (blue arrows) but it is not as clear as the previous examples. Flow in L/min. Delta-P_AOP: Airway pressure from PEEP until AOP. Delta-V_AOP: Volume inside the circuit before AOP. Ccircuit: circuit compliance. Delta-P_Patient: Airway pressure from AOP to the end of the maneuver. Delta-V_Patient: Volume delivered from AOP until the end of the maneuver. Crs: Compliance of the simulated respiratory system.

Table 2: Standard operating procedure for the pediatric AOP maneuver

**Settings**	**Notes**
SIMV-VC<br>Vt = 6 mL/kg of predicted body weight<br>< 20 kg: RR = 10 bpm, Ti = 3 sec<br>20-50 kg: RR = 5 bpm, Ti = 4 sec<br>(Set inspiratory pause at 0)<br>> 50 kg: adult maneuver (Reference 9)<br>PEEP = 5 cmH_2O<br>FiO2 = 1.0	SIMV-VC allows use of previous mode/return to previous settings quickly<br>Long Ti allows for slow insufflation. Low flow makes resistive pressure insignificant.<br>Servo U requires flow adaptation to be OFF (default)
Alarms: Peak pressure alarm 30-35 cmH_2O	This ensure safe limit to the inflation pressure that is used[1]
Disconnect the sampling line of an inhaled nitric oxide (iNO) delivery system. Consider disconnecting the side stream sampling end-tidal CO_2 ($etCO_2$) monitoring	The maneuver is unreliable in the presence of a gas leak[2]
Absence of spontaneous respiratory efforts	Needs passive breathing[3]

VC: Volume control, Vt: tidal volume, RR: respiratory rate, bpm: breath per minute, Ti: insufflation time. Reference 9: The Toronto Centre of Excellence in Mechanical Ventilation (https://coemv.blog/) [Available from: https://respiratorycalc.com/ri-ratio]

1. For safety reasons, even though a Vt of 6 mL/kg is proposed, the high pressure (peak inspiratory pressure) alarm is recommended to be set to 30-35 cmH_2O to ensure excessive pressure is not applied. This step is very important in patients with severe p-ARDS like those who may be supported by extracorporeal membrane oxygenation (ECMO), since an airway pressure of 30-35 cmH_2O can be reached using only a Vt of 1-3 mL/kg secondary to the low Crs. The maneuver is interrupted before delivery of the set Vt if compliance is very low, however if airway closure is present and less than the high-pressure alarm, it will be seen in the pressure tracing of the ventilator, as is displayed in case #2. Alternatively, a lower Vt can be set in patients with very low Crs.

2. The second important point to mention is that this maneuver is unreliable and cannot be performed in the presence of gas leaks, such as active bronchopleural fistula or an uncuffed ETT with detected airway leak. It is also advisable to disconnect side stream sampling end-tidal CO_2 ($etCO_2$) monitoring and the sampling line of an inhaled nitric oxide (iNO) delivery system as the gas sampling acts as a source of leakage.

3. The last requirement is that the patient should not trigger nor have spontaneous respiratory efforts during the length of the maneuver (passive breathing).

Since the minute ventilation will be lower during the duration of the maneuver, the main effect we have observed is a transient increase in $etCO_2$, with no clinical consequences.
